# Instructors for on-the-job training of advanced paramedics – definition of competencies and development of a quality management tool for a "High Responsibility Organization"

**DOI:** 10.3205/zma001216

**Published:** 2019-02-15

**Authors:** Markus Flentje, Deniz Böhmelt, Lion Sieg, Hendrik Eismann

**Affiliations:** 1 Hanover Medical School, Department of Anaesthesiology and Intensive Care Medicine, Hannover, Germany; 2 German Red Cross, Rettungsschule Niedersachsen, Goslar, Germany

**Keywords:** On-the-Job Training, Medicine, Emergency

## Abstract

**Objective: **The psychological demands placed upon the emergency medical services, assures them of their place amongst High Responsibility Organizations. A high pressure to act and an irreversibility of situations are integral features of their workplaces. After the emergency services’ job profiles were restructured in Germany, the practical stage of paramedic training is now undertaken in these conditions. That is, they are trained by a supervising instructor whilst caring for critically ill patients. This paper aims to describe the requisite skills for such an instructor, formulate the associated competences as learning objectives, and develop a quality-measuring instrument for the description of training situations.

**Methods: **The compilation of a competence catalogue was done via a two-step process: following a Delphi survey with an expert panel of practical trainers and trainees, a large cohort of parameters were validated in terms of their relevance. Those factors that formed scales together were identified.

**Results: **After validating the results of the Delphi analysis, six scales (composed of 25 items in toto) were defined. They included the areas of "Training during times of action", "Training during periods of calm", "Background and practical relevance", "Character and personality traits", "Pedagogical competencies" and "Organizational behaviour".

**Conclusion: **For the first time, a competency catalogue has been developed for instructors working in the emergency medical services from German-speaking countries. The catalogue focuses upon clinical training during the acute care of critically ill patients. The scales and items can be used for training-the-trainers, and also quality monitoring. Further research needs to focus on the application of the catalogue in clinical practice and evaluate the need for situational customization.

## Zusammenfassung

**Zielsetzung: **Arbeitspsychologisch wird die Notfallmedizin zu den High Responsibility Organisationen gezählt. Hoher Handlungsdruck und Unumkehrbarkeit der Situation sind einige der Rahmenbedingungen dieses Arbeitsplatzes. Seit einer Neustrukturierung der Berufsbilder im Rettungsdienst, findet nun in dieser Umgebung der praktische Teil der Ausbildung zum Notfallsanitäter statt. Unter Anleitung eines Praxisanleiters erfolgt die Ausbildung während der Versorgung kritisch kranker Patienten. Ziel dieser Arbeit war es, notwendige Kompetenzen eines Praxisanleiters zu beschreiben, um Kompetenzen als Lernziel zu formulieren und ein Qualitätsmessinstrument zur Beschreibung der Ausbildungssituation zu erarbeiten.

**Methodik: **Die Erarbeitung des Kompetenzkataloges erfolgte über ein zweistufiges Verfahren: nach einer Delphi-Befragung mit Praxisanleitern und Auszubildenden wurden die Parameter innerhalb eines großen Kollektivs hinsichtlich der Relevanz validiert. Anschließend wurden Faktoren identifiziert, die zusammen Skalen bilden.

**Ergebnisse: **Nach Validierung der Ergebnisse der Delphi-Analyse ergaben sich sechs Skalen mit insgesamt 25 Items. Sie umfassten die Bereiche „Anleitung im Einsatz“, „Anleitung in der Ruhezeit“, „Hintergrund und Praxisbezug“, „Persönlichkeit“, „Pädagogische Kompetenzen“ sowie „Organisatorisches Verhalten“.

**Schlussfolgerung:** Erstmals wurde für den deutschsprachigen Raum ein Kompetenzkatalog für Ausbilder in der Notfallmedizin entwickelt, der als Voraussetzung auch die Anleitung am Notfallpatient beinhaltet. Die Skalen und Items können für die Ausbilderschulung und für das Qualitätsmonitoring verwendet werden. Weitere Arbeiten müssen sich mit der Anwendung des Kataloges in der Praxis beschäftigen und den Bedarf notwendiger Individualisierung überprüfen.

## 1. Introduction

By virtue of their work environments, the emergency services are counted amongst the various occupations that are collectively described as “High Responsibility Teams” [1]. Amongst other things, work in the emergency services is characterized by time pressure, taking responsibility for the lives of others, an irreversibility of measures taken and limited opportunities for rest- or meal-breaks. In addition to the requirement for specialist knowledge, “non-technical skills” such as communication and task management are requisite factors for the successful handling of situations. The present study evaluates the requirements of a practical vocational training in this specialised work environment. With the enactment of the “Notfallsanitätergesetz” (Advanced Paramedic Law) in January 2014, the new job profile “Advanced Paramedic” was introduced to Germany [https://www.gesetze-im-internet.de/notsang/BJNR134810013.html]. The aim of this amendment was to establish a staff qualification within the emergency services that allows such duly-qualified personnel to provide more advanced measures, including the administration of regular medications, particularly in cases of delays with the emergency doctor’s arrival to the scene.

The advanced paramedic must operate within an established framework of Standard Operating Procedures (SOPs) and algorithms as prescribed by the Medical Director of Emergency Services. The aim of the training is for the autonomous and timely execution of emergency measures in patients who are critically ill. The practical instructor’s task is to steadily lead the trainee to the independent execution of their professional duties. After completing their preliminary training, which usually includes a first year of vocational and practical training, a trainee paramedic will then be paired with an instructor paramedic so as to form a working ambulance crew. Problems can arise as a result of this pairing, namely when the dynamics of an emergency situation precludes supervision (and hence practical instruction) of any measures carried out by the trainee. When an emergency physician is not onsite during these difficult situations, it is the instructor who then carries the burden of responsibility for the patient’s management, including any potential legal consequences. Furthermore, if the patient is transported to hospital by ambulance without an accompanying medical doctor, the task of designating “vehicle guidance” versus “patient monitoring” takes place by individual agreement – again carrying the potential risks of limited trainee supervision. Additionally, generational differences (ranging from social affiliations to attitudes towards leadership and behaviour) pose a potential for conflict [2]. Critical situations, as defined within the framework of High Responsibility Teams, occur more frequently in emergency medical work environments than in other professions – thus a realistic assessment of the trainee’s competencies is required more expeditiously by their instructors.

Following a rise in demand for the newly created job profile, the vocational training was extended to a three-year programme. Training regulations are listed online in the “Training and Examination Regulations for Advanced Paramedics” (Ausbildungs- und Prüfungsverordnung für Nofallsanitäterinnen und Notfallsanitäter: NotSan-APrV) [https://www.gesetze-im-internet.de/notsan-aprv/]. This differentiates between the two categories “theoretical and practical instruction – practical education” and “practical training – chaperoned practice”. The practical training in the emergency services is carried out by specially qualified paramedic instructors (either the “new” advanced paramedics, or paramedics trained prior to 2014 – covered under the §3 NotSan-APrV with a transitional regulation until the end of 2019), who have at least two years of clinical working experience and an additional qualification in Occupational Pedagogy (minimum 200 course contact–hours). Usually, these additional qualifications include continuing education in the areas of “Pedagogy”, “Methodology”, “Media Use” and “Legal Studies”. However, there are currently no fixed regulations in Germany regarding the required course content for these qualifications.

Until now, there have been no guidelines, methods or competencies published that help instructors to delineate or optimize the above described complex situations for themselves and/or their trainees. Expert panels and Delphi analysis have been successfully used as an effective method in the identification of occupational skills in emergency medicine as well as describing target competencies for training students [3] and also for their instructors [4]. However, the Canadian emergency medical services (primarily a paramedic-based service), differ significantly to the German dual emergency care system (emergency physician and paramedics working in tandem at-the-scene) – thus it would be sensible to also perform such studies in the German cultural sphere.

The aim of the present work is to describe the relevant characteristics of and target competences for practical instructors that engender a positive influence upon the trainees’ learning. Additionally, the expected exigencies of day-to-day work-life in the emergency services need to be identified, so as to facilitate instructors with the successful accomplishment of their tasks. The data collected can be used to form the foundations of a training and further education curriculum, as well as a measuring tool of the quality in education. This study project (No. 3600-2017) was reviewed and approved by the Ethics Committee of the Hanover Medical School.

## 2. Methods

A two-stage procedure was used to describe the necessary competencies for practical instructors. In the first stage the essential qualities, as well as their individual characteristics, and competencies were identified by an expert panel using an electronic Delphi procedure (eDelphi). Unfortunately, there is no established universal procedure for performing a Delphi analysis currently [5]. In comparison to previous applications of the eDelphi methodology in emergency medicine [3], our approach differed mainly with regards to cut-off limits used during individual rounds of the survey and also in the number of completed rounds. The current study’s course is described in greater detail below.

**Figure 1:**
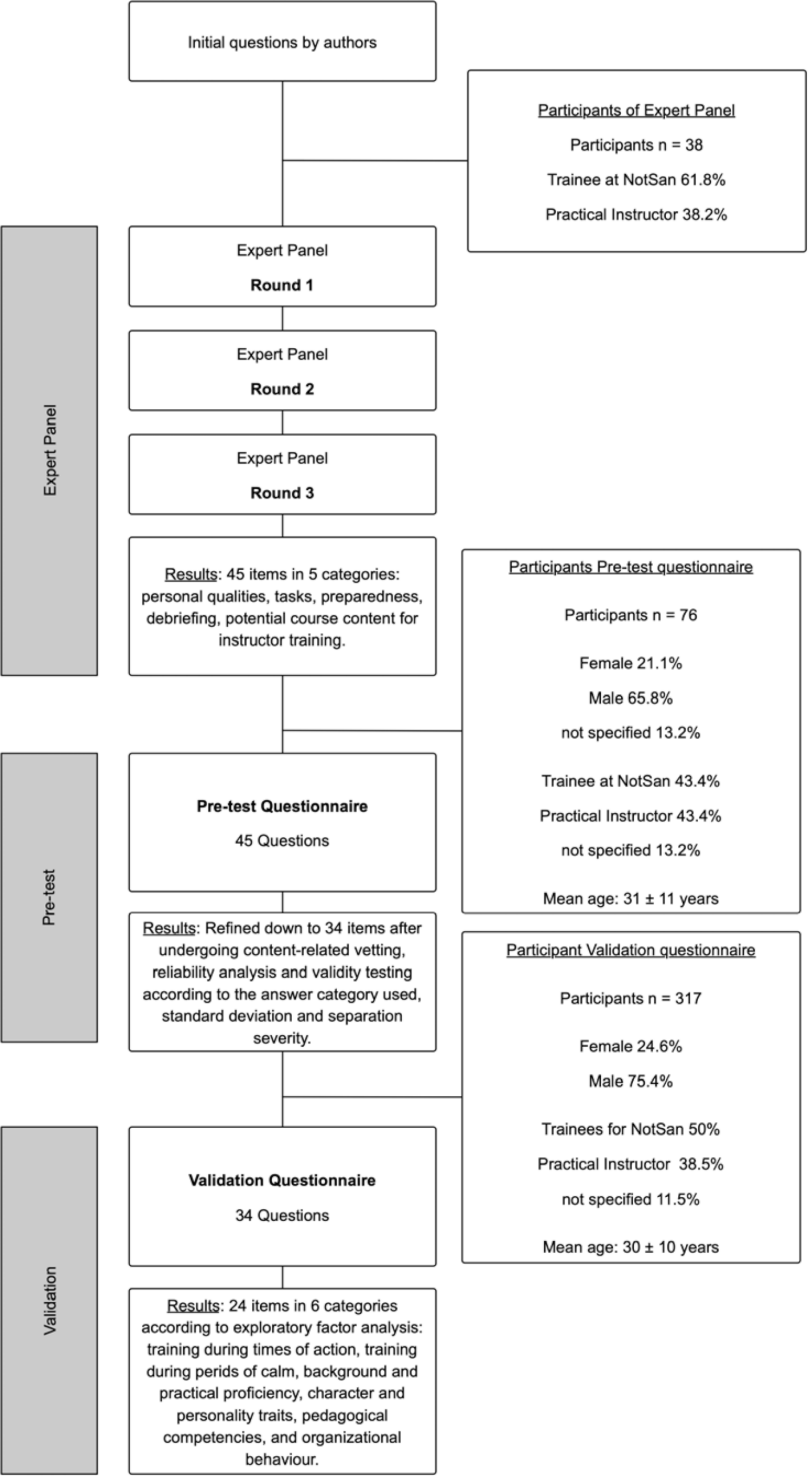
Flow chart of the study process. The demographic data of the study participants are listed in each case before the corresponding questionnaire phase.

During the second stage, parameters were identified from within a large cohort by both practical instructors and trainees, and then validated. Details of the study course are shown in figure 1 (Fig. 1).

### 2.1. eDelphi Procedure

For the first round of the eDelphi analysis, 20 active emergency services instructors and 20 third-year students from an advanced paramedic training class at the German Red Cross Emergency Services School in Lower Saxony were recruited as members of the expert panel. Each question-and-answer session was conducted using the online survey tool “SurveyMonkey” (SurveyMonkey, San Mateo, USA). Every participant had a period of ten days in which to participate in the survey and were given a reminder by e-mail regarding participation in the study. The questionnaire included a greeting, a description of the study objective, a data privacy statement and legal information. All members of the expert panel were asked the same initial questions, which are presented in table 1. These initial questions were allocated by consensus to the items of: personal qualities (Q1, Q2), action during periods of emergency deployment (Q3-Q5) and planning of overall course content (Q6) and are presented in table 1 (Tab. 1). The participants answered the questions as free text during the first round of the eDelphi. During the second round, the participants then received all of the answers tabulated during the first round of questions (the content from several of the answers was condensed by the authors prior to recirculation [6] and were asked to select the items that they considered to be the most relevant. In the third round, participants were asked to select the five most important items. Items that had been selected by at least 25% of the participants were entered into the next round of the eDelphi, or after the third round, incorporated into the pre-test questionnaire.

#### 2.2. Pre-test Questionnaire

Based upon expert opinions and the eDelphi rankings, items with pre-defined answers [7] were developed for the central theme “Qualities of a Practical Instructor”. The resulting questionnaire consisted of 45 items divided into six different categories. The aim was to retain the pertinent content from each selected item whilst developing an efficient and comprehensive pre-test questionnaire. The pre-test questionnaire in its entirety can be found in attachment 1 . A unipolar response scale ranging from 0 to 100 was used for rating each item – allowing for greater test validity and reliability [8], [9]. The participants used a numeric slider to rate the items on a scale from 0 (unimportant) to 100 (very important). To conduct the pre-test, a link to the survey was sent to 38 (actively working) practical instructors and 38 trainees (20 third-year trainees, 18 second-year trainees) from the German Red Cross Emergency Services School in Lower Saxony. With regards to the survey tool, survey period and privacy details, the same procedures were used as during the eDelphi analysis.

The mean and standard deviation were calculated for each item. These results were used for the process of serial item elimination (scale purification), and also to evaluate the responses from the pre-test questionnaire. A reliability analysis was then carried out to evaluate the internal consistency of the individual scales.

#### 2.3. Questionnaire Validation

In regard to the assessment of questionnaires for construct validity, larger sample sizes result in more valid and reliable analyses. Hence, participant recruitment for the validation–questionnaire was done by means of both direct electronic invitations and open invitation (on the social network platforms “Facebook” and “Xing”), with a direct link to the survey being embedded into both invitation styles. The digital recruitment was designed to reach out to all 135 registered advanced paramedic schools in Germany, and specifically targeted their paramedic trainees and paramedic instructors. By performing an exploratory factor analysis, it was possible to deduce the presence of a few underlying latent variables from amongst the observations derived from the large number of items included in the questionnaire. The Kaiser-Guttmann criterion was used to determine the number of factors [10], and an orthogonal varimax rotation was used to gain simplicity in factor interpretation during factor analysis.

All statistical calculations were done with SPSS 24 (IBM Corporation, USA).

## 3. Results

### 3.1. eDelphi

The Delphi analysis was conducted by using eDelphi and was completed after three rounds of question deliberations. The demographic data of the participants are shown in figure 1 (Fig. 1). The initial stage of the Delphi survey subcategorised the items into the central theme “Qualities of a Practical Instructor”. A total of 45 items were generated after completing the final iteration of expert opinions: 18 items were related to personal qualities, 10 on tasks, 9 on the preparedness, 4 on debriefing, and 5 on potential course content for instructor training. Attachment 1 shows the linked answer options for each of the 45 items from pre-test development to the final pre-test questionnaire.

#### 3.2. Pre-test Questionnaire

A total of 76 people participated in the pre-test questionnaire (see figure 1 (Fig. 1)). The first phase of pre-test results’ analysis and item refinement was done by calculating the mean values and normal distributions of the items, as well as checking their content validity. Questions with similar or shared content were then compared, and from these similar items, only those with superior mean and normal distribution values were kept for further consideration. During the second phase, a reliability analysis was performed on the scales to check for internal consistency. It was decided from an economic perspective that the final questionnaire should contain no more than 35 items in total. Consequently, a third phase of analysis was undertaken on the remaining items that rechecked their mean values, standard deviation and normal distribution, whilst ensuring that all response categories had been retained. This three–stage process of question refinement resulted in the elimination of 11 items in total and the creation of a 34–item final questionnaire (see table 2 (Tab. 2)).

#### 3.3. Questionnaire Validation

A larger sample, 317 participants in total (see figure 1 (Fig. 1)), was used during construct validity testing of the refined (34 item) questionnaire to ensure a higher level of validity in the test results. Exploratory factor analysis identified six factors and 25 items from the data – they accounted for 63.4% of the variance. Additionally, a factor load of greater than 0.3 was calculated for the 25 items, which is considered relevant [10]. Sampling adequacy for the factor analysis was determined by use of the Kaiser-Meyer-Olkin (KMO) test, which returned a value of 0.927 – KMO values greater than 0.8 are considered very good [10]. The factors, the resulting Cronbach's alpha, the number of items per factor and the proportion of variance explained are shown in table 3 (Tab. 3). The factors that did not meet the Kaiser-Guttman criterion were not included in the final analysis worksheet. Based upon this criterion, a total of nine items were not included in the final list, including “Professionalism of the instructor” and “Instructor led debriefings”. Moreover, the original scales became redundant and required new categorisations after factor analysis resulted in the creation of new item-groupings. Consequently, the authors defined two new, broad content-based categories for the scales: “the Temporal States and Training” (Training during times of action; and Training during periods of calm) and “the Instructor as a Person” (Background and practical proficiency; Character and personality traits; Pedagogical competencies and Organizational behaviour). The factor “Training during times of action” describes the practical instruction of the trainee during real emergency situations; whereas the factor “Training during periods of calm” refers to the instructor’s availability and tutelage during times of readiness, but not during acute patient care. The factor “Background and practical proficiency” describes to what extent the instructor can combine theoretical and practical knowledge and also fosters the development of trainee competency in both of these fields. The factor “Character and personality traits” assesses an instructor’s behaviours and attitudes towards both the occupation and the team. The factor “Pedagogical competences” relates to the effectiveness of the instructor’s vocational educational and training techniques, the factor “Organizational behaviour” describes the way in which the instructor accomplishes the non-medical aspects of tasks. To translate these separate components into a quality measuring module, the items were transferred to an active formulation. The final item categories and the description of the measured qualities are shown in table 4 (Tab. 4).

## 4. Discussion

The aim of the present work was to describe the necessary competences of a practical instructor in the working environment of a High Responsibility Organization (HRO), and in addition, develop an associated tool for quality measurement. It is known that teacher quality has a significant affect upon the main factors which influence learning in the school environment [11]. Similarly, it is conceivable that learning in a practical setting can be influenced by the trainer’s behaviour. The work environment in emergency medicine would be interesting to study the germane items, since HRO are particularly resistant to influences from the underlying conditions. The Delphi method represents an effective way of performing this analysis, providing a straightforward platform to recruit and interview an expert panel created from trainees and instructors working and training in the recently created profession “advanced paramedic”.

Competency in teaching cannot be taken for granted even amongst teachers who have completed a medical education [12]. Particularly in Anglo-American populations, competence-based models have been developed that provide teachers with conceptual orientations [13]. Since our item list also deals with concrete situations, such as training during emergency services deployment, the results will be discussed in terms of both general competences as well as special competences specific for emergency medical training.

Srinivasan et al. suggest that medical educators should fulfil six core competencies (see table 5 (Tab. 5)) and also described different working environments. These competencies included curriculum development, evaluation, leadership, and practical training.

Based on this concept, the GMA Committee on Personal and Organizational Development in Teaching developed, by means of an incremental consensus process, a position paper on the core competences for medical teachers (KLM), primarily for use in German-speaking countries [14]. Although the overall categories were retained, partial competences were individually adapted for German conditions and their order was rearranged. In comparison to our study, this framework was designed as a universal guide for teachers in medicine, which is reflected by its level of detail. For example, the field “Social and communicative competence” has the learning objective “... willing and able to recognize difficult situations and conflicts and to resolve them constructively”. Whereas our equivalent learning objective was described with more explicit detail: “… neither argue with nor humiliate us in front of the patient”. When comparing our competence fields to those of the KLM (see table 5 (Tab. 5)), all of our items have equivalent practical applications within their field categories. Another item in the KLM is labelled “Creating a learning-friendly atmosphere”, but unfortunately the nature of the basic working conditions in emergency medicine (such as high pressure to act, confusing situations and patient death) cannot always be influenced. While our item “Our practical instructors look after complex assignments” takes this issue into account and further demonstrates the differences between generic competencies and those that provide more specific situational directions. The KLM item “System-related learning and teaching” has limited applicability with the paramedic training situation, since this function is carried out by emergency services staff (not the instructor) and the trainee. Nonetheless, it is the instructor’s duty to update the trainees regarding the current training curricula (not the service staff). Due to the relatively short-term nature of the paramedic practical training path, many emergency service employees are not involved with the trainee’s paramedic training curricula, even though rostering may result in them interacting with each other during normal duties. On the whole, there are no true contradictions between KLM’s and our items, but rather the KLM forms a framework that we refined and fleshed out for a specific environment, creating a more concrete set of assessment criteria.

Thurgur et al. also explicitly investigated the specific situation of training in the emergency environment and identified 14 general principles that promote effective teaching in the field of emergency medicine [5]. Despite underlying geopolitical differences (Canadian versus German emergency services), their findings are in line with ours. They developed the items “Positive teacher attitude” (e.g. motivation), “Treats residents as a colleague” (e.g. respect) and "Provides independence" (e.g. autonomy). Furthermore, items concerning time management (“Use teachable moments well” and “Take time to teach”) were also identified. Additional review of the literature for items related explicitly to the quality of instructors in emergency medicine, also identifies “feedback” and “debriefing” [15] as well as the “teacher quality” in schools [11]. These items were eliminated from our survey after the application of the Kaiser-Guttman criterion during the validation phase. Of note, there is a long tradition for debriefings in the emergency services industry [16], as well as having an established role in education and training – especially simulation teaching methods [17]. Likewise, they are extensively utilized after responses to major disasters [18]. Therefore, debriefings are in actuality an interdisciplinary and pan-professional normality, although our respondents did not find it to be a priority item for this study’s training situation. As with the KLM, our outcomes were congruent with those from Thurgur et al. The loss of individual items, such as debriefings, was partly the result of item-related prioritizations in the context of having set a target for less than 50 items.

Despite inherent structural differences that must exist between training courses or studies undertaken for different occupations, it is in our opinion more than reasonable to compare the literature for emergency paramedic trainings with those of physician medical-trainings. Since both occupations face identical pressures related to the provision of prompt and successful interventions during the care of critically ill patients. Other mutually shared risks include: acting upon false indications, poor implementation of intervention(s), or even incidents beyond the medic’s control (e.g. medication side effects or unavoidable procedural complications). The results show that fostering the individual is desired (“... leads towards the tasks and broadens the competence level”).

It would also be advantageous for practical instructors to gain a firm understanding into their trainees’ backgrounds (including what “Generation” they are represented by), since differences in a target group’s background can also have an impact upon training outcomes.

The generational differences amongst staff working in emergency medical care has previously been described [19]: “Baby Boomers” (1945-1964), “Generation X” (1964-1980), and “Generation Y” (1980-1999) have all been shaped by different social environments and hence also have differences attitudes (e.g. towards hierarchies, or even mobile web usage for obtaining knowledge). The “Big Five” Factor Model [20] describes the dimensions of personality and is applicable to both instructors and learners. The book discusses in detail the characteristics of “the desire for stability”, “extraversion”, “openness”, “willingness to conform” and “conscientiousness”, and their influence on the communication between teachers and trainers – hence also providing practical guidance for work during emergency medical situations. Even though our item “pedagogical competence” has a key competence of communication with trainees, its implementation is primarily geared towards measures influencing the target group as a whole. None of the items we developed are capable of portraying subtleties at an individual level. As such, further research targeting this neglected aspect of trainers in emergency medicine would be desirable and has been urged for by other working groups [21]. The individual personal qualities of both the instructors and their trainees could be a focus for research into these ancillary areas.

## 5. Limitations

Trainings and implementation of measures for the emergency services in Germany is a very heterogeneous affair, since they are administered in a non-central manner by the various federal states. In the authors’ state of Lower Saxony, both the acceptance of advanced paramedics and the implementation of extended measures have spread extensively around the province. Uncertainty in these circumstances may affect instructor-to-trainee ratios in different regional areas. This is exemplified by the matter that their legal certainty is hitherto still under discussion. Nor is it regulated to what extent trainees are allowed to carry out actions under the directive of “extended measures”. In the experience of the authors, measures such as venepuncture and administration of inhaled medications have nonetheless already been incorporated into clinical paramedic practice. Additionally, with the commencement of the “new” three-year traineeship in 2014, the first graduate year of these new advanced paramedics was in 2017. Hence, there is still a paucity of empirical data regarding the new target group, in particular the trainees, and also any resultant changes in the general conditions. It would not be unexpected that additional competencies for practical instructors will emerge after the situation stabilises.

As was described with the creation of the KLM position paper for German-speaking countries, we had difficulties with allocating the items to the scales. As an example, the item “a good practical instructor is reliable” was assigned to the scale “organizational behaviour”, but it could have also fitted with the scale “character and personality traits”. The weighting of individual items was deemed ineffectual, as we surmised that it would be overly affected by different individual evaluations.

## 6. Conclusion

By using expert knowledge in conjunction with a validation process, we have created a catalogue of the requisite competences for practical instructors in the field of emergency medicine, which focuses upon clinical trainings whilst actively caring for critically ill patients. In contrast to existing competence catalogues, trainees were included as part of the consensus process. This emergency medicine training catalogue represents a novelty for German-speaking countries. It can serve as an orientation in the training of practical instructors and also represents a quality measuring instrument for training of emergency medical services during their routine operation. Ongoing use and evaluation of the catalogue in clinical practice will show whether its introduction improves the quality of training, and that both trainees and practical instructors achieve greater levels of satisfaction.

## Competing interests

The authors declare that they have no competing interests. 

## Supplementary Material

Categories of the pre-test questionnaire with their items. The aim of the pre-test questionnaire was to assess the relevance of the items.

## Figures and Tables

**Table 1:**
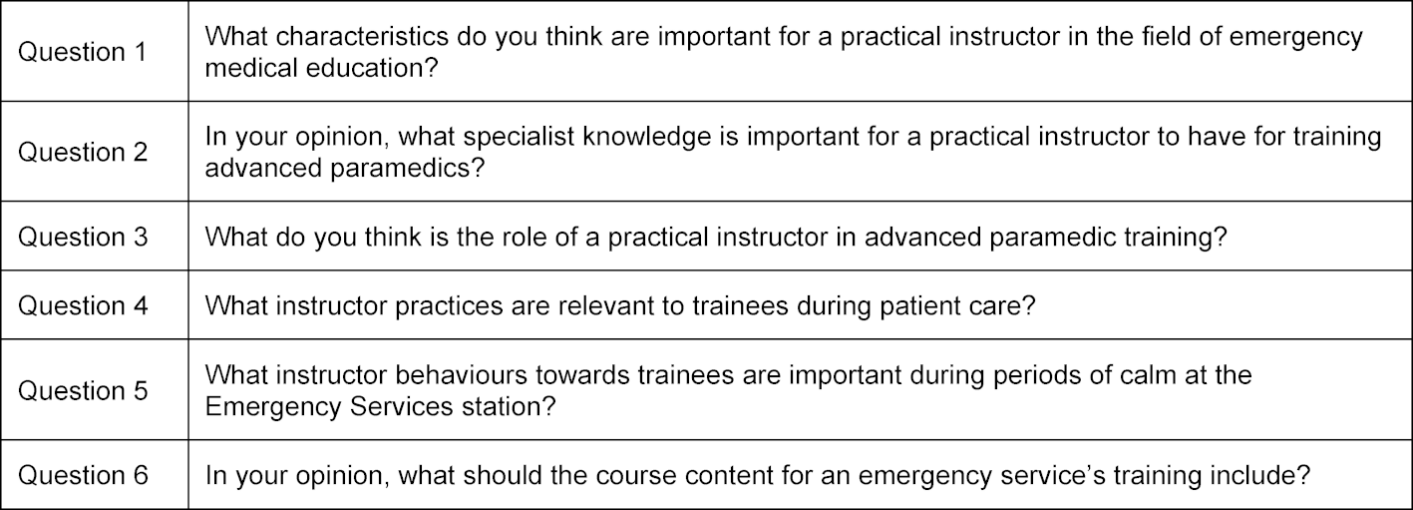
Initial survey questions submitted to the expert panel at the start of the eDelphi analysis.

**Table 2:**
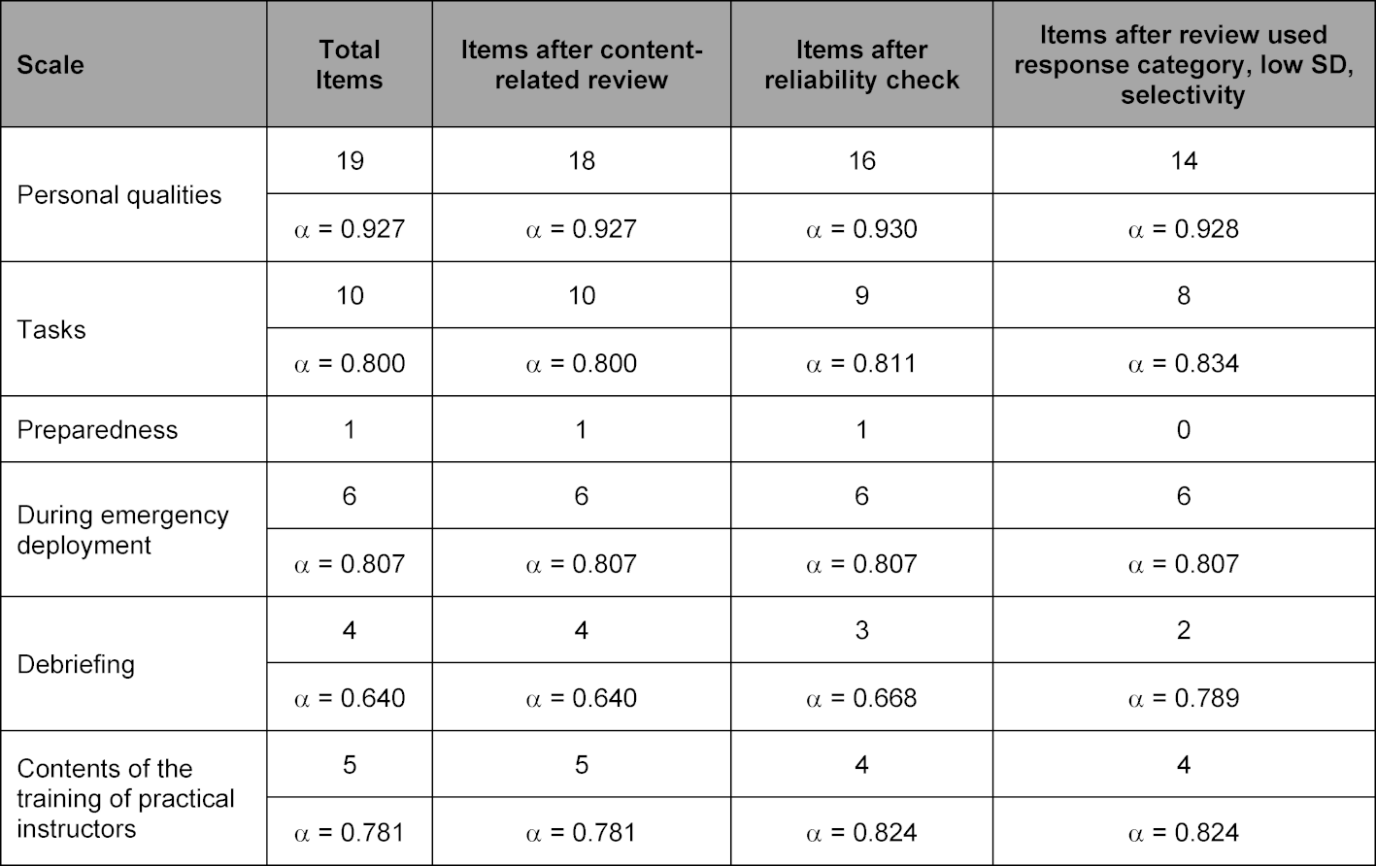
Reduction of items by scale from the pre-test questionnaire to the final questionnaire. Shown are the number of items and Cronbach's Alpha for each scale. SD: standard deviation, α: Cronbach’s Alpha

**Table 3:**
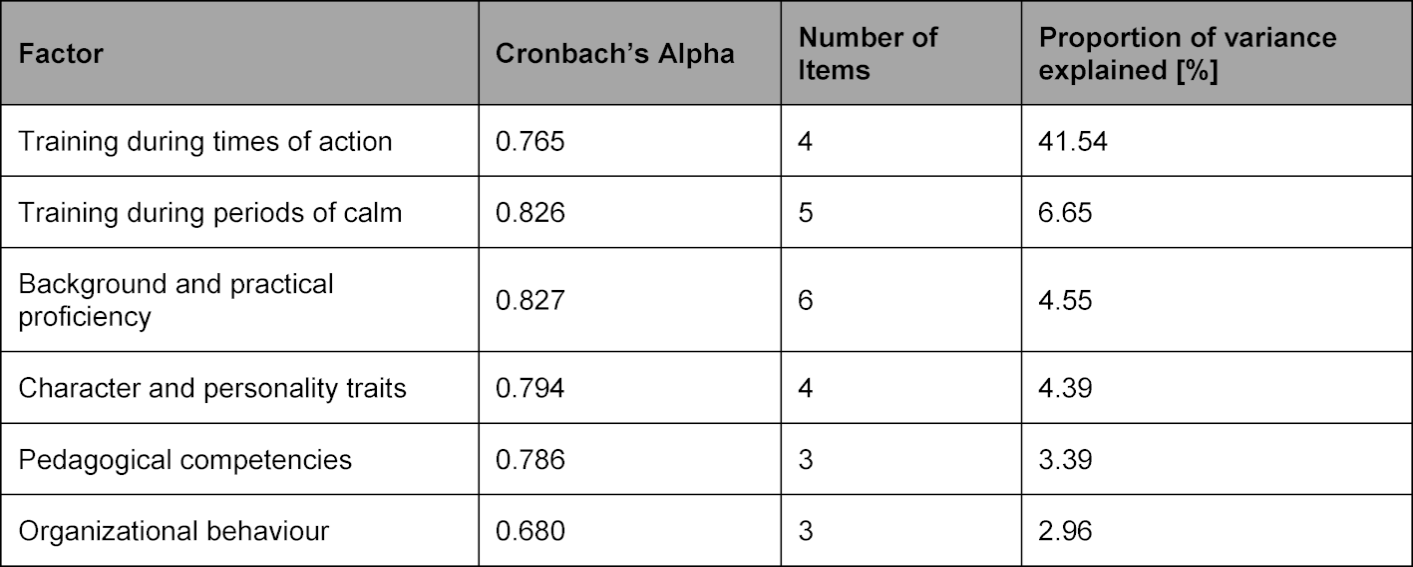
Factors after exploratory factor analysis (with varimax rotation). Shown are the factors, Cronbach's alpha, the number of items per factor, and the variance explanation of the individual factors.

**Table 4:**
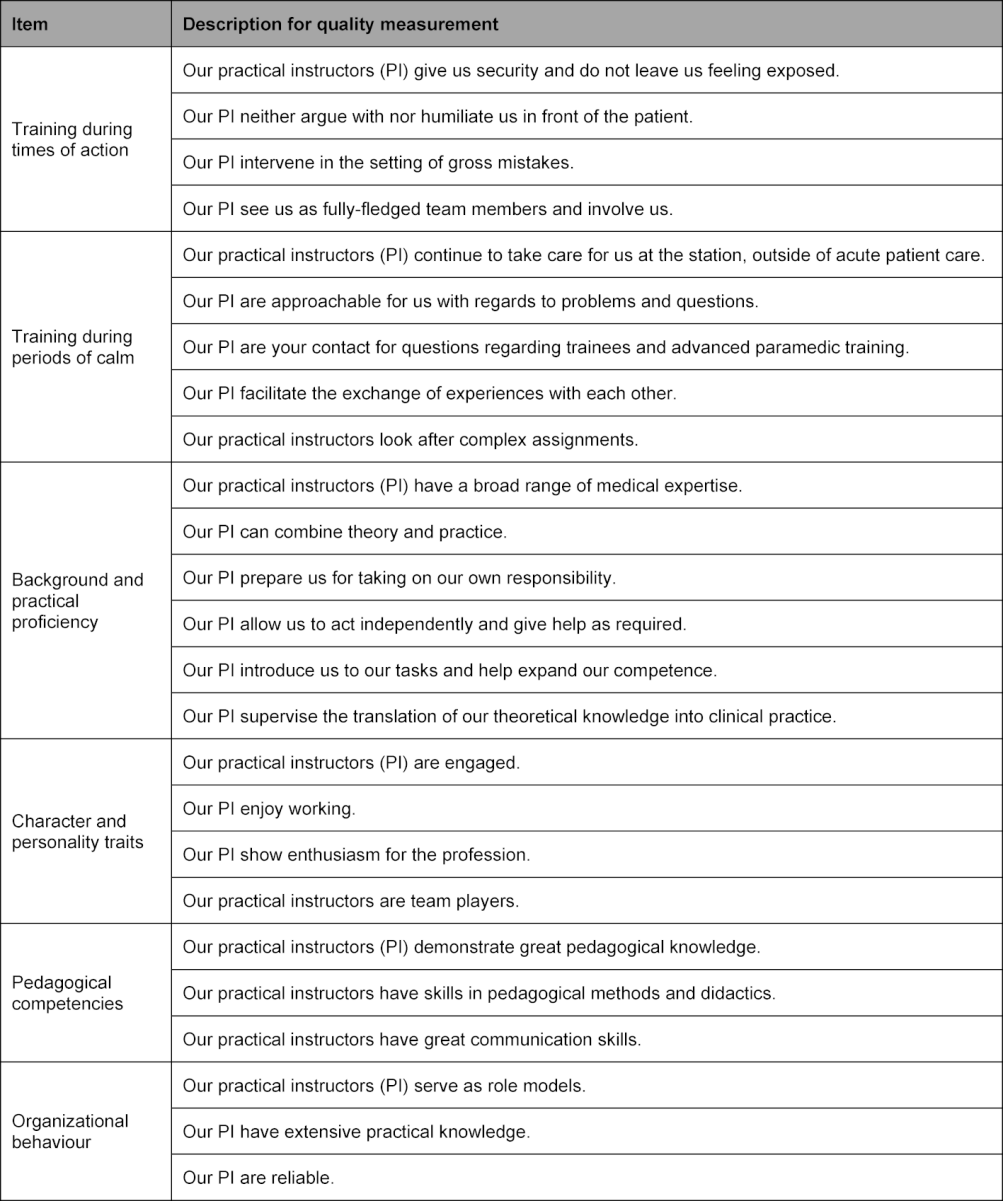
Final categories, items and formulation for quality measurement of competencies for practical instructors. For the quality measuring instrument, the competences were formulated as an active statement. PI: practical instructor

**Table 5:**
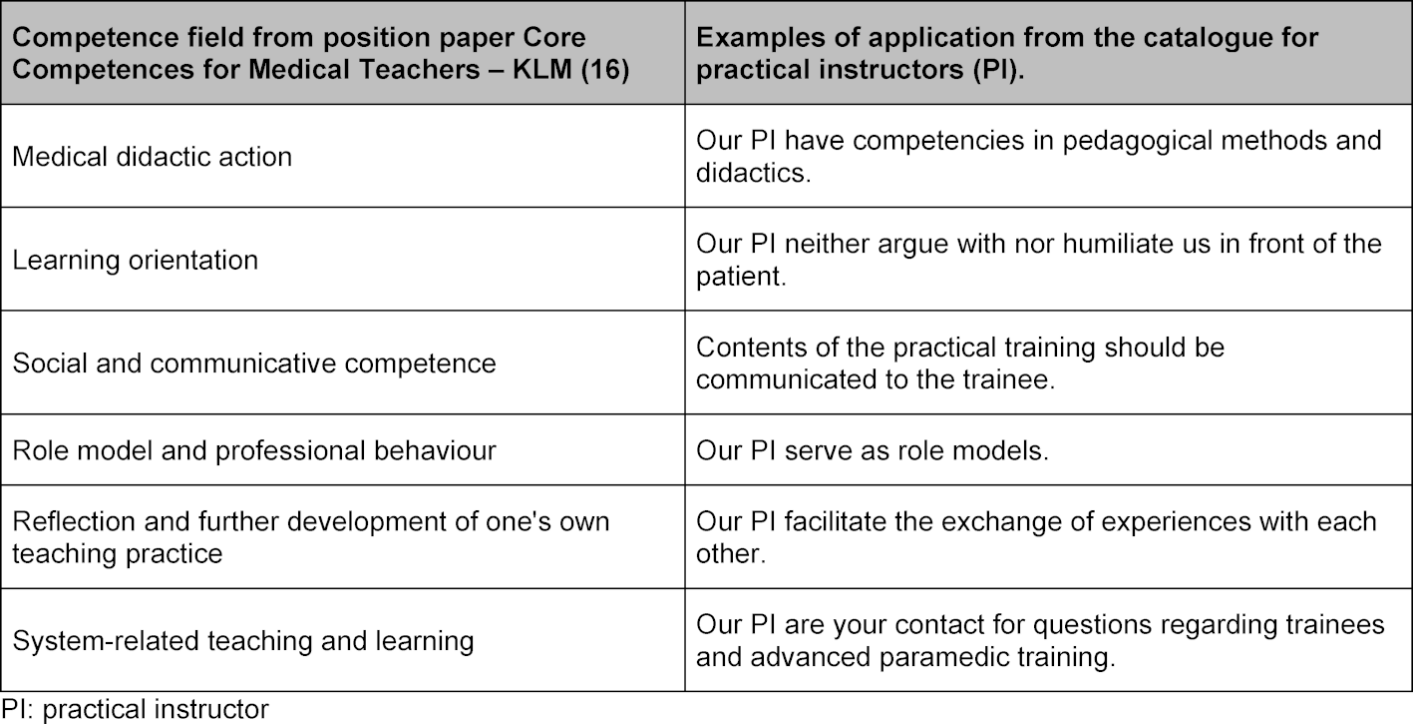
Comparison of the categories and items developed between the core competences of the teachers and our results for practical instructors.
