# Supplementary material for: Instructors for on-the-job training of advanced paramedics – definition of competencies and development of a quality management tool for a "High Responsibility Organization"
Source: GMS J Med Educ. 2019 Feb 15;36(1):Doc8. doi: 10.3205/zma001216 (PMC6390088; doi:10.3205/zma001216)
Supplement: Categories of the pre-test questionnaire with their items. The aim of the pre-test questionnaire was to assess the relevance of the items. [file JME-36-8-s-001.pdf]

Scale	Example Item
Personal qualities	A good practical instructor functions as a role model.
	A good practical instructor has a great organisational knowledge.
	A good practical instructor has a great pedagogical knowledge.
	A good practical instructor knows the training curriculum for critical care paramedics.
	A good practical instructor knows what the Not-San training requirements are.
	A good practical instructor has an extensive medical knowledge.
	A good practical instructor knows the current treatment algorithms (e.g. the NUN-algorithm in Lower Saxony, Germany).
	A good practical instructor knows the current educational doctrine for the critical care paramedic schools.
	A good practical instructor is engaged/motivated.
	A good practical instructor has fun at work.
	A good practical instructor has the ability to reflect.
	A good practical instructor has significant clinical experience.
	A good practical instructor shows enthusiasm for the profession.
	A good practical instructor behaves professionally.
	A good practical instructor is reliable.
	A good practical instructor is empathetic.
	A good practical instructor displays calm and composure.
	A good practical instructor is a team player.
	A good practical instructor is interested in the trainee.
Tasks	A good practical instructor has the task of linking theory and clinical practice.
	A good practical instructor has the task of monitoring the implementation of theory during emergency care.
	A good practical instructor respects the rest periods of the trainee.
	A good practical instructor is also available during idle times to answer questions.
	A good practical instructor has the task of looking after the trainee at the emergency services station.
	A good practical instructor is the trainee‘s contact when problems and questions arise.

	A good practical instructor is your contact for questions regarding trainees and critical care paramedic training.
	A good practical instructor has the task of teaching practical skills.
	A good practical instructor has the task of preparing the trainee for taking personal responsibility.
	A good practical instructor has to take the lead during rescue efforts.
**Preparedness**	A good practical instructor practice at the ambulance station with equipment from his own emergency service.
**During emergency deployment**	A good practical instructor provides surety and does not merely poses.
	A good practical instructor neither argues with nor humiliates the trainee in from of patients.
	A good practical instructor intervenes in the setting of gross mistakes.
	A good practical instructor sees the trainee as a fully-fledged team member and involves them.
	A good practical instructor lets the trainee act independently but provides assistance when required.
	A good practical instructor introduces the trainee to their tasks in the field and expands their competence.
**Debriefing**	A good practical instructor will conduct debriefings.
	A good practical instructor conducts debriefings after onerous missions.
	A good practical instructor conducts debriefing sessions during idle times to work through deficiencies.
	A good practical instructor recognises deficits and addresses them.
**Contents of the training of practical instructors**	The practical instructor‘s training should include pedagogical methods and didactics.
	The practical instructor‘s training should include how to communicate with the trainee.
	The practical instructor‘s training should include the sharing of experiences with practical instructors.
	The practical instructor‘s training should include techniques for the implementation of practical training with the trainees.
	The practical instructor‘s training should include the first discourse after difficult assignments.
